# Circulating cell-free nucleosomes as biomarker for kidney transplant rejection: a pilot study

**DOI:** 10.1186/s13148-020-00969-4

**Published:** 2021-02-11

**Authors:** Jeroen G. H. P. Verhoeven, Carla C. Baan, Annemiek M. A. Peeters, Marian C. Clahsen-van Groningen, Daan Nieboer, Mariëlle Herzog, Marc Eccleston, Dennis A. Hesselink, Karin Boer

**Affiliations:** 1 Department of Internal Medicine, Division of Nephrology and Transplantation, Erasmus MC, University Medical Center Rotterdam, Room Na-524, P.O. Box 2040, 3000 CA Rotterdam, The Netherlands; 2 Rotterdam Transplant Group, Erasmus MC, University Medical Center Rotterdam, Rotterdam, The Netherlands; 3 Department of Pathology, Erasmus MC, University Medical Center Rotterdam, Rotterdam, The Netherlands; 4 Department of Public Health, Erasmus MC, University Medical Center Rotterdam, Rotterdam, The Netherlands; 5 Belgian Volition SPRL, Isnes, Belgium

**Keywords:** Biomarker, Circulating cell-free nucleosomes, Human, Kidney, Rejection, Transplantation

## Abstract

**Background:**

There is an unmet need for noninvasive markers specific for kidney transplant rejection. Such a marker may eventually overcome the need for a transplant biopsy. In this pilot study, the potential of circulating cell-free nucleosomes (CCFN) to serve as a biomarker for kidney transplant rejection was evaluated.

**Methods:**

Forty de novo kidney transplant recipients were prospectively followed as part of a randomized, controlled clinical trial. Total CCFN (H3) and CCFN with the histone modifications H3K36me3 and H3 citrulline were measured in patients at four fixed time points: before transplantation and on days 3–6, 30 and 180 after kidney transplantation. In addition, serum collected at times of transplant rejection (*n* = 14) was analyzed. CCFN were measured with a Nu.Q™ Assay kit (VolitionRx), an ELISA-based assay using antibodies directed against nucleosomes.

**Results:**

For total CCFN (H3), H3K36me3, and H3 citrulline, the same pattern was seen over time: Concentrations were elevated shortly after transplantation (day 3–6) followed by a decline reaching baseline (pre-transplantation) values at days 30 and 180. At times of acute rejection, the median concentration of total CCFN (H3) was significantly higher compared to the stable situation (day 30): 4309 (3435–5285) versus 2885 (1668–3923) ng/mL, *p* < 0.05, respectively. Total CCFN (H3) had an acceptable ability to discriminate rejection from no rejection (AUC-ROC = 0.73) with a negative predictive value of 92.9%. For both histone modifications (H3K36me3 and H3 citrulline), there was no significant difference between episodes of acute rejection and the stable situation (day 30).

**Conclusion:**

In this pilot study, total CCFN (H3) concentrations are increased at times of acute kidney transplant rejection. The high negative predictive value implies that whenever a patient experiences loss of renal transplant function and the total CCFN (H3) is not increased, causes other than acute rejection should be considered. Clinical implementation of total CCFN (H3) measurement may avoid unnecessary and potentially harmful kidney transplant biopsies.

## Background

Approximately 10–20% of kidney transplant recipients experiences acute rejection (AR) within the first year after transplantation [1]. Rejection is associated with an increased risk of (long-term) graft failure and death and accurate and timely diagnosis of acute rejection is important to start anti-rejection therapy as soon as possible [2].

The current diagnosis of acute rejection relies on serial monitoring of serum creatinine, urinary protein excretion and histopathological examination of a needle biopsy of the allograft. However, these parameters have several limitations. Serum creatinine is relatively insensitive to diagnose rejection; a rise in serum creatinine occurs after substantial kidney tissue injury has occurred [3]. In addition, other causes of graft injury, such as infection and drug toxicity, can also lead to increased serum creatinine concentrations or increased urinary protein loss. A needle biopsy to confirm acute rejection is, however, an invasive procedure, has high costs, suffers from sampling error, has a relatively long turnaround time, and is subject to inter-observer variability [4]. In addition, a kidney transplant biopsy is not always feasible, for example, in patients that require anticoagulant therapy and young children [5]. Therefore, there is an unmet need for reliable and minimally invasive biomarkers to diagnose kidney transplant rejection [6]. A candidate biomarker should be cost-effective, give reproducible outcomes, have a short turnaround time, and have a high sensitivity, specificity and positive and negative predictive value. Currently, several biomarkers are under investigation, including biomarkers that reflect injury to the allograft (for example, donor-derived cell-free DNA, kidney injury molecule 1, and neutrophil gelatinase-associated lipocalin) [6–10]. Investigation of other potential biomarkers, including nucleosomes, is necessary as the diagnostic performance of the proposed biomarkers to replace the current diagnostic measures is unclear.

As a result of cell damage, nucleosomes (internal cellular content) are released in the circulation, so-called circulating cell-free nucleosomes (CCFN). Nucleosomes consist of DNA wrapped around histone proteins and are critically involved in the epigenetic regulation of gene expression (6). Histone modifications, such as methylation, acetylation, ubiquitination, and phosphorylation, mark the “tail” domains of histones. These modifications alter the affinity of histone proteins for DNA and thereby control the transcription of genes [11]. Variation in the epigenetic signature of histones determines their cell-specific phenotype and enables discrimination between normal and pathological cells. Total CCFN concentration is increased during several diseases, which make their use as specific biomarker for disease limited [12]. However, specific histone modifications (or combination of these modifications) may allow discrimination between diseases, such as cancer, and normal physiology [13]. In pancreatic cancer patients, combination of the measurement of a DNA modification (5-Methylcytosine) and histone modifications (H2AZ, H2A1.1 and H3K4Me2) with the conventionally used biomarker carbohydrate antigen 19-9 gave a better diagnostic performance for detecting pancreatic cancer than the measurement of carbohydrate antigen 19-9 alone [14]. In the context of acute kidney transplant rejection, serial CCFN values may change as a result of the rejection process. As total nucleosome concentrations generally reflect cell damage, specific nucleosome modifications could serve as markers specific for kidney rejection. Citrullination of histone H3 (H3 citrulline) has been associated with inflammation and with the activation and release of neutrophil extracellular traps (NETs) [15, 16]. Torres-Ruiz et al. showed that these NETs play a role in kidney transplant rejection; higher amounts of circulating NETs were found in patients with AR [17]. Therefore, CCFN with H3 citrulline could be a valuable marker in terms of AR. H3K36me3 has been associated with various types of malignant tumors; altered levels of H3K36me3 have been reported in breast cancer, gliomas and renal cell carcinoma [18–20]. Up until now, only associations has been made with renal cell carcinoma, and not with other types of pathology of kidney tissue. In the present pilot study, we investigated whether total CCFN (H3) and CCFN with specific histone modifications (H3K36me3 and H3 citrulline) in serum of kidney transplant recipients could serve as a biomarker for acute kidney transplant rejection.

## Results

### Study design

**Fig. 1:**
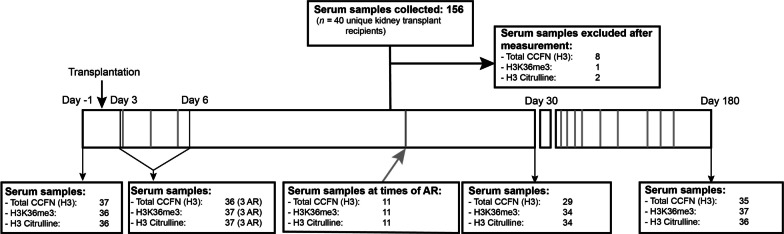
Overview of the study and sampling. The gray lines represent samples collected at times of AR. AR, acute rejection

This study comprised serum samples (*n* = 156) from 40 kidney transplant recipients which were collected at the following time points: 1 day before transplantation and at day 3–6, 30 and 180 after transplantation and at the time of a clinically indicated transplant biopsy (Fig. 1). The baseline characteristics and clinical information after transplantation of these patients are depicted in Table 1. A total of 14 serum samples were measured from biopsy-proven acute rejection episodes, occurring in 11 patients (two patients suffered from 2 or 3 episodes of biopsy-proven acute rejection). Total CCFN (H3) and CCFN with the modifications H3K36me3 and H3 citrulline were separately investigated, and dependent on these measured epigenetic features, 1–8 samples were excluded because of technical failure.

**Table 1:** Baseline characteristics (at time of transplantation) and clinical information

	Study group (*n* = 20)	
Age, y	54 (21–76)	
Male/Female	30 (75%)/10 (25%)	
Etnicity		
Caucasian	33 (82.5%)	
African	4 (10%)	
Asian	3 (7.5%)	
Cause of end-stage renal disease		
Diabetes mellitus	10 (25%)	
Hypertension	7 (17.5%)	
IgA nephropathy	4 (10%)	
Polycystic kidney disease	6 (15%)	
Obstructive nephropathy	4 (10%)	
Unknown	5 (12.5%)	
Other	4 (10%)	
Renal replacement		
Preemptive/ non-preemptive	18 (45%)/22 (55%)	
No. Kidney transplantation		
First	39 (97.5%)	
Second	1 (2.5%)	
Living donor/deceased donor	40 (100%)/0 (0%)	
Immunosuppressive therapy		
Belatacept based	20 (50%)	
Tacrolimus based	20 (50%)	
Biopsy-proven acute rejection (BPAR)	11 (27.5%)	
Time to first BPAR (within the first 6 months after transplantation), days	56 (3–152)	

Continuous variables are presented as medians (including ranges) and categorical variables as numbers (including percentages). Baseline characteristics and clinical information are adapted from de Graav et al. [21]

### Longitudinal analysis of CCFN

**Fig. 2:**
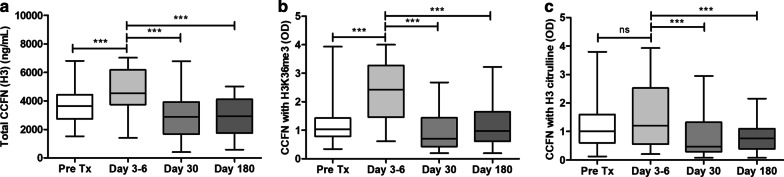
Course of CCFN values in 40 kidney transplant recipients (**a**). Depicted are the concentrations of total CCFN (H3) and ODs of **b** CCFN with H3K36me3 and **c** CCFN with H3 citrulline over time. OD, optical density. N.B.: CCFN box whiskers represent minimal and maximal values. The middle line of the box represents the median and the upper and lower borders represent the 25 and 75% percentile, respectively (*p < 0.05, **p < 0.01, ***p < 0.001)

The values of CCFN of all patients were compared over time at the scheduled time points, except of values measured at times of AR (Fig. 2). For total CCFN (H3), H3K36me3, and H3 citrulline, the same pattern was seen: elevated values shortly after transplantation (day 3–6), followed by a decline and return to baseline values at day 30 and day 180. For total CCFN (H3), the median concentration before transplantation was 3634 (2737–4438) ng/mL serum (Fig. 2a). Total CCFN (H3) concentrations were significantly higher at day 3–6; 4535 (3746–6170) ng/mL (*p* < 0.001). Thereafter, the concentrations returned to baseline: 2885 (1668–3923) and 2921 (1739–4112) ng/mL for day 30 and day 180, respectively (*p* < 0.001).

For H3K36me3, the median OD before transplantation was 1.04 (0.78–1.43) which increased to 2.43 (1.46–3.27) at day 3–6 (*p* < 0.001) (Fig. 2b). Compared to day 3–6, the OD decreased to 0.71 (0.43–1.44) at day 30 and 0.97 (0.62–1.65) at day 180, respectively (*p* < 0.001). For the modification H3 citrulline, the median OD before transplantation was 1.01 (0.60–1.60) compared to 1.21 (0.56–2.53) in day 3–6 specimens (*p* = ns) (Fig. 2c). Compared to day 3–6, the OD decreased at days 30 and 180 to 0.48 (0.30–1.32) and 0.76 (0.39–1.10), respectively (*p* < 0.001).

### CCFN and kidney transplant rejection

**Fig. 3:**
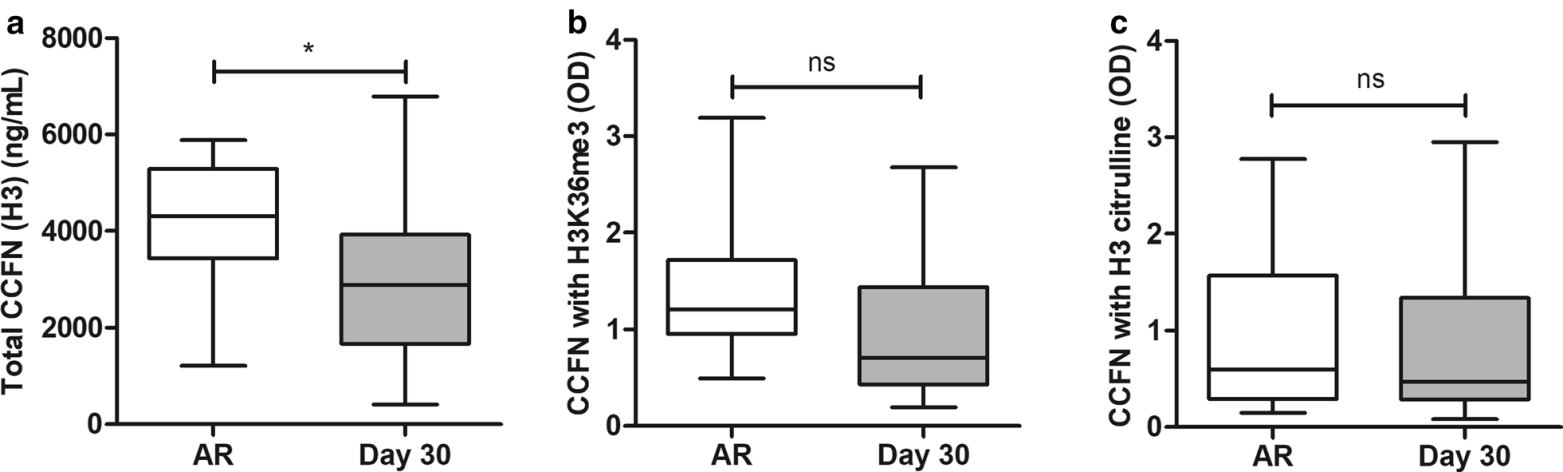
CCFN values at times of AR compared to the stable situation (day 30). **a** Concentrations of total CCFN (H3) and **b** ODs of CCFN with H3K36me3 and **c** H3 citrulline. OD, optical density. The middle line of the box represents the median and the upper and lower borders represent the 25 and 75% percentile, respectively (*p < 0.05, **p < 0.01, ***p < 0.001)

Next, CCFN values during AR were studied. To this end, samples with AR were compared to stable samples without AR (collected at day 30). During AR, the median concentration of total CCFN (H3) was with 4309 (3435–5285) ng/mL significantly higher compared to 2885 (1668–3923) ng/mL in samples without AR (Fig. 3a; *p* < 0.05). These results demonstrate an increase of 49.4% at times of AR compared to the stable situation.

For both histone modifications, no significant differences were found between samples with AR and stable samples without AR. For H3K36me3, the median OD at times of AR was 1.21 (0.95–1.72), compared to 0.87 (0.56–1.50) during no AR (*p* = ns) (Fig. 3b). For H3 citrulline, the median OD at times of AR and no AR were 0.60 (0.29–1.57) and 0.67 (0.32–1.16), respectively (*p* = ns) (Fig. 3c).

**Fig. 4:**
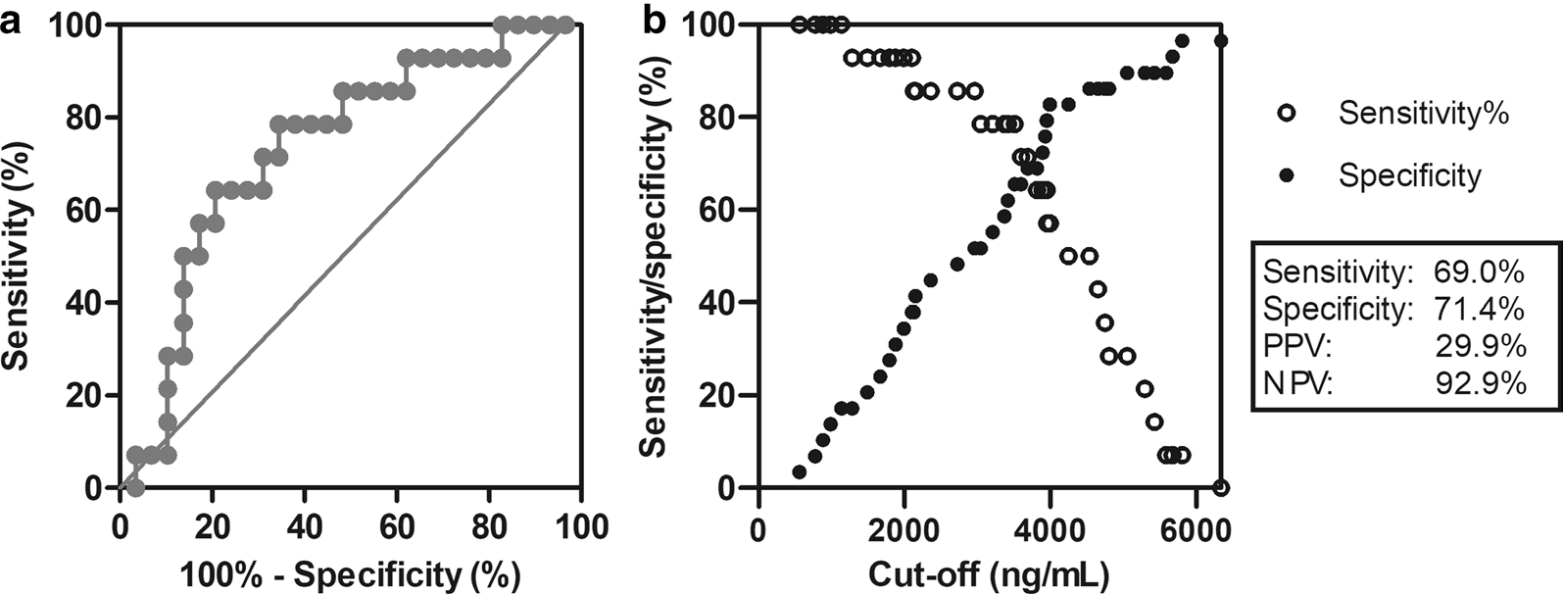
Diagnostic performance of total CCFN (H3) to discriminate from the stable situation (day 30 and day 180). **a** ROC curve and **b**. Sensitivity (black) and specificity (white) depicted over the observed range of CCFN concentrations. Reported sensitivity and specificity correspond to a cutoff of 3687 ng/mL serum. PPV and NPV are based on an incidence of AR of 15%. AUC, area under the curve; PPV, positive predictive value; NPV, negative predictive value

Next, the performance of total CCFN (H3) to discriminate AR from samples without AR was investigated by an AUC-ROC analysis. An AUC of 0.73 (95%-CI, 0.62–0.85; Fig. 4a) was calculated, showing a 69.0% sensitivity (95%-CI, 50.8–82.7) and 71.4% specificity (95%-CI, 45.4–88.3) with a cutoff of 3687 ng/mL, to discriminate AR from no AR (Fig. 4b). The corresponding PPV and NPV for total CCFN (H3), based on an incidence of AR of 15%, were 29.9% and 92.9%, respectively.

## Discussion

This pilot study was designed to investigate the potential of CCFN as a biomarker for the detection of acute rejection after kidney transplantation. The main finding is that total CCFN (H3) concentrations are increased at times of acute rejection compared to no acute rejection. For H3K36me3 and H3 citrulline, no significant differences were found between episodes of rejection and no rejection. Total CCFN (H3) had an acceptable ability to discriminate rejection from no rejection. In particular, the NPV of total CCFN (H3) is high. This means that this test can potentially avoid a risky and costly kidney transplant biopsy in a patient with a deterioration of kidney function. This may result in fewer biopsy-related complications such as (life-threatening) bleeding or the formation of arteriovenous fistula [4]. Monitoring CCFN concentrations can especially be beneficial in patients who cannot undergo a biopsy (as a result of the need to continue anti-coagulant therapy) or in small children who require general anesthesia when performing a biopsy. Finally, CCFN monitoring can help to reduce the number of transplant biopsies in patients with potentially more harmful biopsy-related complications, such as in cardiac transplant recipients.

In contrast to the high NPV, the low PPV for total CCFN (H3) is an obvious limitation. However, we feel that even when the PPV of total CCFN (H3) would have been higher, this still would not circumvent the need for a biopsy. A biopsy is not only necessary to demonstrate acute rejection but also discriminates different rejection types which require different therapies [22, 23].

The present findings demonstrate that both histone modifications are not associated with acute rejection in kidney transplant recipients. Nevertheless, it could still be possible to increase the specificity of nucleosomes as a marker for kidney transplant rejection by measuring other nucleosome modifications.

Another finding of this study was that CCFN values change over time. Both total CCFN (H3) and CCFN with H3K36me3 were significantly higher shortly after transplantation (day 3–6) and total CCFN (H3) and both modifications (H3K36me3 and H3 citrulline) decreased significantly at the day 30 and day 180 time points. Increased values shortly after transplantation have also been observed for other potential biomarkers for acute rejection, such as circulating cell-free DNA [6, 24]. We speculate that ischemia and repair and regeneration processes (accompanying kidney transplantation) that results in ischemia–reperfusion injury (IRI), leads to increased CCFN values shortly after transplantation due to cellular apoptosis and necrosis [25].

Despite the relatively few rejection events (*n* = 14), limiting the statistical power of this study, significant differences were observed. Three of these rejections occurred at day 3–6 after transplantation when CCFN may have increased as a result of IRI. Currently, we are collecting samples in a larger cohort of kidney transplant recipients where the effect of IRI on CCFN during rejection should be explored by comparing rejections occurring shortly after transplantation with rejections occurring later on after transplantation. It would also be of interest to investigate CCFN using ImageStream(X)-based imaging technology, a newly identified promising methodological approach to measure CCFN [26].

The rejections in this study were all biopsy-proven, whereas the no rejection samples were not. This means that within the no rejection group, serum samples may have been collected at times of a subclinical rejection, which by definition, can only be detected by protocol biopsy as patients with a subclinical rejection do not have clinical graft dysfunction. To rule out the possible effect of subclinical rejection, it would be preferable to perform protocol biopsies, which allows for the comparison of biopsy-proven rejection samples with biopsy-confirmed no rejection samples.

An advantage of CCFN is that the measurement can be performed within hours, requires very small amounts of sample volume (10 µL), and that large variety of different measurable histone modifications are available [14]. This large variety allows for the epigenetic profiling of CCFN to identify specific combinations of epigenetic features which can be used to improve the diagnostic performance of CCFN to serve as a marker for acute rejection. It would be interesting to assess the discriminatory capacity of CCFN to differentiate between different types of acute rejection and between rejection and other types of pathology such as acute tubular necrosis, BK virus nephropathy, pyelonephritis and renal calcineurin inhibitor toxicity.

## Conclusion

Analysis of CCFN concentrations in serum of kidney transplant recipients is a promising minimally invasive diagnostic tool to screen for acute rejection after kidney transplantation. Measurement of total CCFN (H3) has a high negative predictive value for acute rejection. These findings suggest that whenever a patient experiences loss of renal transplant function and CCFN is not increased, causes other than acute rejection should be considered. Clinical implementation of CCFN measurement may avoid unnecessary and potentially harmful transplant biopsies.

## Methods

### Patient population and samples

Serum samples were collected from 40 kidney transplant recipients who participated in an investigator-initiated, prospective, randomized-controlled, single-center, clinical trial performed at the Erasmus MC, University Medical Center, Rotterdam, the Netherlands [21]. The study was approved by the Medical Ethical Review Board (MERB number METC-2012-421) and was registered in the Dutch national trial registry (https://www.trialregister.nl/trials; number NTR4242, registered October 2013). All participating patients gave written informed consent before inclusion. Samples were collected at the following time points: 1 day before transplantation and at day 3–6, 30, and 180 after transplantation and at the time of a clinically indicated transplant biopsy (collected 4 days before, till 1 day after the biopsy) (Fig. 1). Only serum samples of patients who were diagnosed with biopsy-proven acute rejection (as opposed to other histopathological diagnoses) were analyzed. No protocol biopsies were obtained in this trial.

The serum samples were centrifuged (1910 g, 10 min) and the supernatant was stored at − 80 °C until further use. All biopsies were scored independently by two experienced kidney pathologists according to the Banff 2015 classification [27]. At the time of the trial, donor-specific anti-HLA antibodies were not routinely measured and therefore the biopsies could not be scored according to the most recent Banff classification [28].

### Cell-free nucleosome immunoassays

Total CCFN (histone H3) and CCFN with the modifications H3K36me3 (commonly altered in several cancer types) and H3 citrulline (induced in neutrophils in response to inflammatory stimuli) were separately measured by the manufacturer using a not commercialized NuQ® ELISA (Belgian Volition SPRL, Isnes, Belgium) according to the manufacturer’s instructions [14, 29]. The ELISA assays used were sandwich ELISA’s where (1) the capture antibody consisted of an antibody raised again either a histone H3 epitope for the quantification of CCFN (H3) or against H3K36Me3 or H3 citrulline, for the two nucleosome modification assays, respectively; (2) the detection antibody was a biotinylated anti-nucleosome detection antibody directed against a nucleosome conformational epitope (which confirms that nucleosomes were measured and not histones). In brief, 96-well microtiter plates were coated with a capture antibody and 10 μL of serum was dispensed into each well (in duplicate). After incubation for 150 min, the biotinylated anti-nucleosome antibody solution was added to each well and the plate was incubated for another 90 min. Wells were then washed with a wash buffer (provided by Volition), a streptavidin–horseradish peroxidase solution was added and the plate was incubated for 30 min. Subsequently, wells were washed, substrate solution was added and after 10–20 min, the colorimetric reaction was stopped by adding the diluted stop solution (provided by Volition). All incubation steps were performed at 15–25 °C with orbital shaking at approximately 700 rpm. The optical density (OD) of the wells was determined with a spectrophotometer at 405 nm. For both histone modifications, values were expressed as OD units, while for the total CCFN (H3) values were expressed as concentration (ng/mL serum).

### Statistical analysis

All analyses were performed using GraphPad (5.01, GraphPad, Inc, LA Jolla, CA) and SPSS version 25.0 (IBM Corp., Armonk, NY, USA). Continuous variables are reported as median and inter-quartile range (IQR; first and third quartiles). To evaluate CCFN values over time, samples at different time points from the same patients were compared using a linear mixed model with a random intercept for patient to adjust for repeated measurements within patients.

To evaluate the performance of CCFN as a biomarker for acute kidney transplant rejection, samples were divided into an AR group and a non-AR group. The non-AR group consisted of day 30 samples of clinically stable patients who were free from (biopsy-proven) AR at this time point, or from other causes of (histologically confirmed) allograft injury. The AR group consisted of samples of patients with biopsy-proven rejection. In this group, all samples collected from different rejection events were used for the analysis. Differences between the AR measurements and non-AR measurements were compared using a linear mixed model with a random intercept for patient to adjust for multiple measurements per patient.

The area under the curve–receiver operating characteristic curve (AUC-ROC) was used to assess the discriminative ability of total CCFN (H3) for differentiating AR and non-AR. Based on a data-driven cutoff value, sensitivity and specificity were calculated. The positive predictive value (PPV) and negative predictive value (NPV) for AR were calculated based on an assumed incidence of AR of 15%. The 95% confidence intervals of the AUC were estimated using bootstrapping. All statistical tests were considered statistically significant when the two-sided *p* value was below 0.05.

## Data Availability

Data can be shared upon written request to the corresponding author.

## Abbreviations

AR: Acute rejection
AUC-ROC: Area under the curve-receiver operating characteristic
BPAR: Biopsy-proven acute rejection
NETs: Neutrophil extracellular traps
CCFN: Circulating cell-free nucleosomes
NPV: Negative predictive value
PPV: Positive predictive value
